# Comparative analysis of pulmonary and extrapulmonary tuberculosis of 411 cases

**DOI:** 10.1186/s12941-015-0092-2

**Published:** 2015-06-24

**Authors:** Aysel Sunnetcioglu, Mahmut Sunnetcioglu, Irfan Binici, Ali Irfan Baran, Mustafa Kasım Karahocagil, Muhammed Rıdvan Saydan

**Affiliations:** Department of Pulmonary, Yuzuncu Yil University Medical Faculty, Van, Turkey; Medical Faculty, Department of Clinical Bacteriology and Infectious Diseases, Yuzuncu Yil University, Van, Turkey; Department of Clinical Bacteriology and Infectious Disease, Ercis State Hospital, Van, Turkey; Tuberculosis Control Dispensary, Van, Turkey

**Keywords:** Tuberculosis, Pulmonary, Extrapulmonary, Epidemiology

## Abstract

**Background:**

Tuberculosis is a disease that can involve every organ system. While pulmonary tuberculosis is the most common presentation, extrapulmonary tuberculosis (EPT) is also an important clinical problem. The current study aimed to outline and compare the demographic and clinical features of pulmonary and extrapulmonary tuberculosis cases in adults.

**Methods:**

Medical records of 411 patients (190 women, 221 men) treated between January 2010 and July 2014 in provincial tuberculosis control dispensary was retrospectively reviewed. Demographic and clinical characteristics were compared for pulmonary and extrapulmonary tuberculosis cases.

**Results:**

Of these 411 cases, 208 (50.6 %) had pulmonary tuberculosis (PTB) and 203 were diagnosed with extrapulmonary tuberculosis (EPTB) (49.4 %). The average ages for PTB and EPTB groups were 33.00-27.00 and 31.00-29.75, respectively (*p* = 0.513). Men were more frequently affected by PTB (59.6 %), while EPTB was more commonly detected in women (52.2 %) (*p* = 0.016). Main diagnostic modalities for PTB were sputum/smear analyses (72.7 %), clinical-radiological data (21.7 %) and biopsy (6.1 %); while biopsy (71.5 %), sputum/fluid analysis (18.8 %) and clinical-radiological data (4.9 %) were used for confirming EPTB (*p* < 0.0019). The most common sites of EPTB involvement were lymph nodes (39.4 %), followed by pleura (23.6 %), peritoneum (9.9 %) and bone (7.4 %).

**Conclusıons:**

Extrapulmonary involvement of tuberculosis is common and females are more likely to be affected. Increased clinical awareness is important since atypical presentations of the disease may constitute diagnostic and therapeutic challenges.

## Background

Tuberculosis (TB) is a chronic, granulomatous, bacterial infection that may display multisystemic involvement. It still constitutes a major global health problem and it is estimated that almost one-third of the population is infected worldwide [1, 2]. Main microbiological agent is *Mycobacterium tuberculosis* in the vast majority of cases [2]. The disease may transform into the active phase in 10 % of cases and lungs are the most common site of involvement [1–3].

Pulmonary tuberculosis (PTB) is a highly contagious infection that may disseminate in the initial period after infection [2, 3]. The proportion of PTB to EPTB varies with respect to geographical, social, ethnic and economical parameters [3]. The rate of extrapulmonary tuberculosis (EPTB) patients in Turkey is estimated as 39 %-45.1 % [1].

Owing to a functional immune system, the foci of infection formed after the initial infection may be silent in the beginning. However, the disease may be reactivated at anytime and anywhere in the body [4]. This reactivation may be enhanced by the immune compromisation and EPTB may become clinically manifest in this setting [4, 5].

The most common sites for EPTB are lymph nodes, pleura, cutaneous tissue, abdomen, gastrointestinal system and bones [1, 3, 4]. Attributed to its atypical presentation, diagnostic difficulties, increasing prevalence and potential to result in hazardous sequelae, increased clinical awareness for EPTB is crucial [2, 5]. Diagnosis should be established immediately and treatment must be started to reduce the morbidity and mortality due to EPTB.

The current study was conveyed to outline and identify the demographic and clinical characteristics of PTB and EPTB comparatively. Thereby, we hope to figure out clinical clues may be determined for better recognition and more effective management of disease.

## Material and methods

### Study design

Medical records of the patients treated between January 2010 and July 2014 in provincial tuberculosis control dispensary was retrospectively reviewed in accordance with the principles of the Helsinki Declaration. Ethics committee approval was obtained for this study. Demographic and clinical data were extracted from the medical files of 411 cases diagnosed with active tuberculosis. Age, gender, site of involvement, recurrence rate and diagnostic methods were recorded and compared in PTB and EPTB patients. Patients were divided age groups as intervals of <21, 21-40, 41-60 and >60 years.

Diagnosis of PTB and EPTB are made in accordance with definitions of World health Organization [6]. Pulmonary tuberculosis was ruled in if two or three initial sputum analyses were positive for acid-fast bacilli (AFB) or one sputum smear positive for AFB in conjunction with clinical and radiological data consistent with tuberculosis. Diagnosis of EFTB was established if fine needle aspiration biopsy or biochemical analyses of pleural/ascetic or other fluid samples or other histopathological examinations yielded relevant results.

Exclusion criteria consisted of immune deficiency, usage of immunosuppressive medications, simultaneous pulmonary and extrapulmonary involvement by TB and age < 15.

### Statistical analysis

Analysis of data was made via the IBM Statistical Package for Social Sciences (SPSS) 20 program. The conformability of the data to the normal distribution was tested with Kolmogorov-Smirnov test. Parameters that display normal distribution were evaluated with parametric methods, whereas variables without a normal distribution were assessed by non-parametric methods. Mann-Whitney *U* test was used to compare 2 independent groups and comparison of categorical variables was carried out using Pearson’s chi-squared test exact method. Since age variable did not exhibit normal distribution, it was expressed as median-interquartile range. Confidence interval was set at 95 % and level of statistical significance was set at p <0.05.

## Results

The whole study population consisted of 203 (49.4 %) EPTB and 208 (50.6 %) PTB patients. The median-interquartile ranges for PTB and EPTB groups were 33.00-27.00 and 31.00-29.75, respectively. Two groups were similar in terms of age distribution (*p* = 0.513).

Analysis of data after allocation of cases in age groups as intervals of <21, 21-40, 41-60 and >60 years was made. In EPTB group, males were more frequently affected in patients younger than 21 years and females were more commonly involved in the other three age groups (*p* = 0.034). In contrast, women were more likely to be susceptible in patients <21 years and men were predominantly affected in the remaining 3 age groups in PTB (*p* = 0.007) (Table 1).

**Table 1:** Distribution of age and sex in PTB and EPTB groups

Age groups (years)		EPTB	*p* Value		PTB	*p* Value
	Female	Male		Female	Male	
	(*n*,%)	(*n*,%)		(*n*,%)	(*n*,%)	
<21	16 (37 %)	28 (63 %)	0.034*	22 (58 %)	16 (42 %)	0.007*
21-40	51 (55 %)	42 (45 %)		33 (38 %)	54 (62 %)	
41-60	25 (57 %)	19 (43 %)		11 (23 %)	36 (77 %)	
>60	14 (58 %)	10 (42 %)		18 (50 %)	18 (50 %)	

* statistically significant

Women were more commonly affected by EPTB (106/203; 52.2 %), while men (124/208; 59.6 %) were predominantly involved in PTB group (*p* = 0.016). Rate of recurrence and number of patients re-admitting after interruption of treatment was similar in two groups (*p* = 0.172 and *p* = 0.97). Diagnostic modality used for confirmation of TB was different in two groups: For EPTB group, biopsy was the main mode (71.5 %) followed by sputum/smear analyses [18.8 %], for PTB group whereas sputum/smear analyses was the most frequent method (72.7 %) succeeded by clinical and radiological diagnosis (21.7 %) (*p* < 0.001). Positivity for BCG scar was similar in both PTB and EPTB groups (*p* = 0.491). Overview of these demographic and clinical data is shown in (Table 2).

**Table 2:** Comparison of parameters under investigation in PTB and EPTB groups

Variable		EPTB	PTB	*p* Value
Age (years)	<21	42 (20 %)	38 (18.2 %)	0.513
	21-40	93 (45.8 %)	87 (41.8 %)	
	41-60	44 (24.6 %)	47 (22.5 %)	
	>60	24 (11.8 %)	36 (15.8 %)	
Gender	Female (n, %)	106 (52.2 %)	84 (40.4 %)	0.016*
	Male (n,%)	97 (47.8 %)	124 (59.6 %)	
Recurrence rate (*n*,%)		4 (1.97 %)	9 (4.26 %)	0.172
Re-admission after interruption of treatment		3 (1.48 %)	3 (1.44 %)	0.97
Presence of BCG scar		123 (59.6 %)	117 (56.3 %)	0.491
Most common modes of diagnosis	Biopsy (*n*,%)	103 (71.5 %)	11 (6.1 %)	<0.001*
	Smear (*n*,%)	27 (18.8 %)	129 (72.7 %)	
	C&R clues (*n*,%)	7 (4.9 %)	39 (21.7 %)	

*: statistically significant; *EPTB* extrapulmonary tuberculosis, *PTB* pulmonary tuberculosis, *C&R* clinical and radiological

The most common sites were lymph nodes (39.4 %), pleura (23.6 %), peritoneum (9.9 %) and bone (7.4 %) (Table 3).

**Table 3:** Sites of involvement in EPTB

Site	Number (%)
Lymph nodes	80 (39.4 %)
Pleura	48 (23.6 %)
Peritoneum	20 (9.9 %)
Bone	15 (7.4 %)
Meninges	11 (5.4 %)
Urinary system	11 (5.4 %)
Breast	7 (3.4 %)
Pericardium	4 (2 %)
Other sites (GI tract, eye, etc.)	7 (3.4 %)

*EPTB* extrapulmonary tuberculosis, *GI* gastrointestinal

The most common systemic disorders accompanying EPTB were DM (n = 9; 0.04 %) and chronic renal failure (n = 8; 0.04 %), while chronic renal failure (n = 9; 0.04) and chronic obstructive pulmonary disease (n = 6; 0.03 %) were those detected frequently in PTB group. Systemic diseases detected in PTB and EPTB groups are shown in (Table 4).

**Table 4:** Systemic diseases encountered in EPTB and PTB patients

Systemic disease	Group	
	EPTB (number, %)	PTB (number, %)
Diabetes mellitus	9 (0.04 %)	2 (0.01 %)
Chronic renal failure	8 (0.04 %)	9 (0.04 %)
COPD	3 (0.01 %)	6 (0.03 %)
Hypertension	3 (0.01 %)	4 (0.02 %)
Congestive heart failure	3 (0.01 %)	2 (0.01 %)
Lymphoma	3 (0.01 %)	1 (<0.01 %)
HIV positivity	-	1 (<0.01 %)

*EPTB* extrapulmonary tuberculosis, *PTB* pulmonary tuberculosis, *COPD* chronic obstructive pulmonary disease

## Discussion

In this study, we aimed to investigate and compare the demographic and clinical characteristics of EPTB and PTB. Our results indicated that EPTB is almost as common as PTB and it is more likely to affect women. Moreover, both PTB and EPTB appear as diseases affecting young adults in provincial tuberculosis control dispensary.

The incidence of EPTB was remarkably high (49.4 %) in the present study. The most common sites of involvement were lymph nodes, pleura, peritoneum and bone. Interestingly, men were more likely be affected by PTB and women were more prone to EPTB. Biopsy was the most useful mode of diagnosis in EPTB, while sputum/smear analyses was the most commonly used diagnostic tool for PTB.

Earlier studies performed in different regions of Turkey showed that the rates of EPTB cases among all TB cases ranged from 3.2 % to 53.8 % [7–11]. The predilection of EPTB for women may be linked with the limited facilities for access to healthcare and prevalence of other risk factors such as smoking habit [2, 5]. Smoking is less common in women and this may be one of the factors responsible for the difference in distribution. Moreover, Lam et al. have reported that the risk of mortality increased with smoking in men, but not in women [4, 12]. These results imply that females may be relatively protected against the hazardous pulmonary effects of smoking. In contrary, PTB was encountered more frequently in men (59.6 %) and the higher prevalence of smoking among men in Turkey may be responsible for this finding. However several studies investigated the impact of gender on the occurrence of EPTB and found that women were more likely to present their active TB as EPTB [13–15].

We observed that young adult population was affected by PTB and EPTB. However, Musellim et al. have reported that age was not associated with EPTB [4]. In the past decade, some cohort studies have reported an increased percentage of EPTB in patients with TB [13, 14]. Even though TB is expected to arise more commonly in older population with deterioration of immune system, this finding reminds that control programs must be focussed especially on young population. Campaigns and efforts must be directed to increase public awareness on this topic to combat with contagion of TB.

Similar to most of the other publications, lymph nodes were the most common sites involved in EPTB [1, 2, 5]. Controversially, Yang et al. have reported that bone and joints were involved more commonly in EPTB [16], while another study suggested that genitourinary system and skin were affected more often than lymph nodes in EPTB [17]. These variations may be linked with social or environmental factors . In our series, peritoneal involvement was the third most frequent site for EPTB group in our series.

Pulmonary tuberculosis may present as atypical pneumonia, pleuritis, or upper lobe involvement. It may present in an atypical fashion in case it is associated with HIV [4].

Even though sputum/smear analysis and clinical/radiological clues are mostly beneficial in PTB; biopsy and histopathological diagnosis is usually required for ruling in EPTB [2, 3]. Increased clinical awareness must be followed by choice of appropriate diagnostic method in order to make the diagnosis timely. It must be remembered that *M. tuberculosis culture* is the gold standard for establishing the definitive diagnosis [5]. However, rates of culture positivity are far less than expected not only in PTB cases but also in EPTB group [5]. This circumstance may ensource from limitations of technical facilities. Therefore, other diagnostic modes such as biopsy, sputum/smear analysis and clinical-histopathological data may compensate for the aforementioned restrictions attributed to culture results.

In the literature, risk factors for EPTB were female gender in Asian and North African population, age for sub-Saharan African people and HIV infection in Europe [18]. These risk factors may show variability in different populations and the characteristics of EPTB need to be studied in multicentric studies on larger populations.

Some restrictions of the current study must be mentioned. This study was performed on a small sample size with a retrospective design including only adult patients. In addition, impacts of social, ethnic, economic and environmental factors must be taken into account during extrapolation of our results to larger populations. Owing to the retrospective design, some critical data such as nutritional status, microbial factors and habits such as smoking and alcohol consumption may not be fully achieved from the medical files.

In conclusion, EPTB is a frequent manifestation of TB which has substantial morbidity. Despite atypical features at presentation, diagnosis must be made without delay and appropriate treatment must be started as soon as possible for reduction of morbidity and mortality rates. Tuberculosis control programs must be targeted at specific populations under risk with special care on young and female populations.
